# Carbon Reduction of the Three-Year Air Pollution Control Plan under the LEAP Model Using a GREAT Tool in Panzhihua, China

**DOI:** 10.3390/ijerph192114482

**Published:** 2022-11-04

**Authors:** Junjie Wang, Yi Zhang, Linde Mei, Xuemei Xu, Hanmei Yin, Xiaoqiong Feng, Junhui Chen

**Affiliations:** 1 Sichuan Academy of Environmental Sciences, Chengdu 620041, China; 2 School of Environment, Sichuan University, Chengdu 610065, China; 3 School of Environment, Tsinghua University, Beijing 100084, China

**Keywords:** carbon emissions, LEAP model, energy, carbon reduction

## Abstract

In the context of global warming and climate change, various international communities have set different reduction targets for carbon emissions. In 2020, China proposed that CO_2_ emissions will peak by 2030 and reached a critical period in which carbon reduction is a key strategic direction. Sichuan Academy of Environmental Sciences published the “Panzhihua Three-Year Iron Fist Gas Control Action Plan” in 2021. The measures implemented in the plan only address general considerations of conventional pollutants in the atmosphere. This study established the Panzhihua LEAP model based on the GREAT tool and built four simulation scenarios, including pollutant treatment upgrade (PTU), traffic improvement (TI), boiler remediation (BR), and baseline scenarios for industrial sources, mobile sources, and industrial boilers in policy implementation. It provided a supportive basis for the development of environmental protection measures in Sichuan province to increase the efficiency of carbon emission reduction. The quantitative analysis of the simulation results for the five years from 2020 to 2024 was conducted to discuss the intrinsic links between carbon emissions and energy consumption, market storage, and demand under different scenarios. It concluded that the BR and TI scenarios benefit carbon reduction, while the PTU scenario negatively impacts it. This study provided recommendations for analyzing the carbon footprint at a city-wide level, quantifying the relationship between the implementation of relevant environmental measures and carbon emissions, which are available for policy development that incorporates carbon reduction considerations and offers relevant support for future research.

## 1. Introduction

With the continuous development of economic and social development and the acceleration of globalization, the emission of greenhouse gases such as CO_2_ in the atmosphere has increased significantly, making climate change one of the most serious challenges faced by mankind worldwide today, which has aroused the universal concern of the international community [1,2]. From the current economic development of various countries, the mainstream developed countries, small and medium-sized economies with mainly service industries, and countries with relatively stagnant industrial development have achieved carbon peaking [3]. Germany, Norway, the Czech Republic, and Romania achieved peak carbon in 1990, the United States and Australia achieved it in 2007, and Japan completed its target in 2013 [4]. In 2020, China proposed to peak CO_2_ emissions by 2030; carbon peaking was included in the government work report in 2021. China has entered a critical period in which carbon reduction is the key strategic direction, promoting synergy and efficiency in reducing pollution and reducing carbon emission [5].

Panzhihua City is the largest mining city in the upper reaches of the Yangtze River in China and one of the typical industrial cities [6]. According to the data survey statistics of previous years, Panzhihua City also ranks first and third in the world in terms of Ti and V reserves at the same time [6]. In the last half century, after large-scale rapid economic development, Panzhihua City experienced rapid economic growth based on mining development. However, environmental problems have also become increasingly prominent [7]. According to the decomposition of air quality improvement targets in Sichuan Province’s 14th Five-Year Plan, and taking into account the changes in air quality in Panzhihua City over the years, in 2021, Sichuan Academy of Environmental Sciences prepared the PTCP (Panzhihua Three-Year Air Pollution Control Action Plan) [8,9] for the Panzhihua government, which focuses on industrial sources, motor vehicles, and other sources of pollution, and makes a planning route for the development of air environmental protection in Panzhihua City over the next three years. The PTCP only targets sustained improvement in air quality without assessing carbon emissions. Combined with the lack of consideration of the impact of carbon emissions in the Sichuan air pollution prevention and control plan up to 2021 [8,9], it is instructive to conduct a carbon emissions effectiveness study on the PTCP as a strategic approach to reducing pollution and carbon emissions in Sichuan.

Models for medium and long-term modeling of carbon emissions are currently standard in academia, such as Yale University’s DICE model [10], which has a dynamic intertemporal optimization mechanism for decision making and response to emission reduction measures. However, it still falls short in describing related processes such as technological progress [11]. The MIT’s (Massachusetts Institute of Technology) EPPA model [12], which often lacks simulation of future carbon emission pathways due to data limitations, is not applicable to city-level carbon emission analysis [13,14]. Priambodo et al. [15] used the LEAP model to analyze the energy consumption and CO_2_ emissions of industrial enterprises in Indonesia and concluded that carbon emissions in the industrial sector in developing countries need to be controlled and focused. Fei [16] analyzed the energy consumption and air pollutant emission levels of transport in Xiamen based on the LEAP model. They concluded that air pollutants and carbon dioxide have a strong homology in the transport sector. Shahida et al. [17] developed a LEAP model for electricity and energy in a typical city in Pakistan, explored the applicability of clean energy options, and concluded that prioritizing local resources could reduce the energy crisis and environmental impacts. Although the measures of switching from coal to new energy sources are expected to reduce carbon emissions in principle, the actual possible impact in China under the current measures, such as industrial boiler retrofit and large-scale replacement of new energy-heavy vehicles, remains unclear [15,16,17].

Combined with the above considerations, this study uses the LEAP model for carbon emission prediction analysis of environmental protection measures in the PTCP to assess the contribution of air pollution prevention and control measures for standard industrial and mobile sources in Panzhihua City to reduce local carbon emissions, and in this way extends to general governance issues such as energy over-consumption. This study effectively assessed the carbon reduction effectiveness of the current mainstream emission control measures for industrial and mobile sources. It provided strong research support for the environmentally friendly measures formulation in Sichuan Province under the carbon reduction conditions.

## 2. Methodology

In this study, the LEAP (the Low Emissions Analysis Platform) model was used to assess the carbon reduction effect of the air management plan of Panzhihua City over five years, from 2020 to 2024. Using 2019 as the base year, the study conducts a midterm simulation analysis of the PTCP’s atmospheric optimization policies on industrial and mobile sources through the LEAP model, dividing the simulation scenarios into four scenarios, including the base scenario, end-of-pipe treatment, boiler coal conversion, and transportation optimization, to obtain the trend of carbon emission changes in Panzhihua City in the next five years on a 100-year GWP (global warming potential) scale.

### 2.1. Models

**Figure 1:**
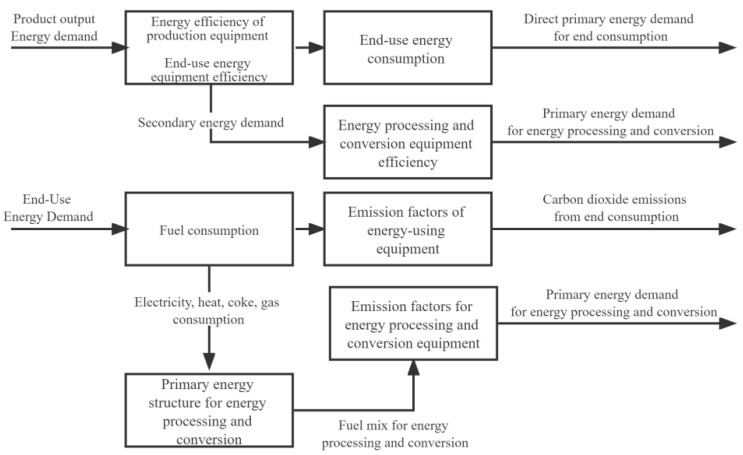
LEAP model technical route [19].

The LEAP model is an effective tool for energy policy analysis and climate change mitigation assessment developed by the Stockholm Environment Institute in Sweden [17]. The LEAP model has been used in more than 150 countries worldwide [16,17], and the technical approach to the model is shown in Figure 1. The model is equipped with modules for energy demand forecasting and environmental impact prediction and analysis, which allow the construction of energy supply and demand scenarios based on critical scenarios of economic, industrial, or technological development and the prediction of future short- or medium- and long-term energy demand based on the actual local conditions of the target population, as well as the prediction of the environmental impact of a given energy scenario based on a compiled environmental database, and the calculation of greenhouse gas emissions from the perspective of resources, transformation, and utilization, and the measurement of greenhouse gas contributions on a 100- and 200-year GWP scale [18,19].

**Figure 2:**
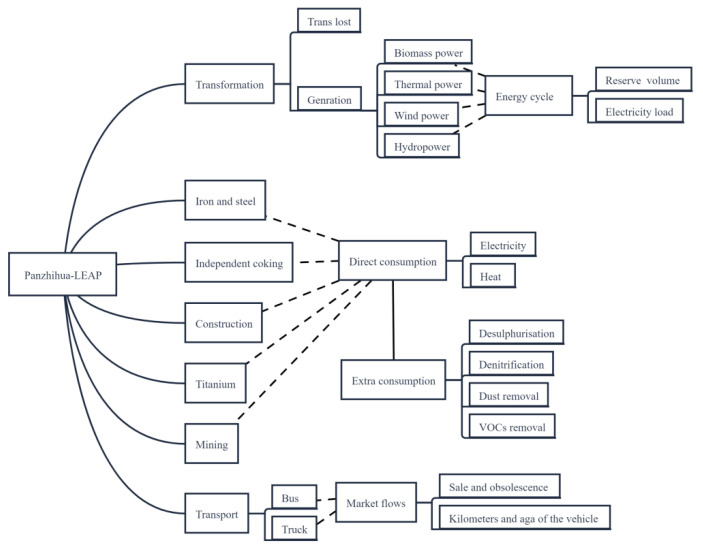
Structure of the Panzhihua LEAP model (this figure was constructed for this study).

GREAT (Green Resources and Energy Analysis Tool) is a LEAP application tool designed by the China Energy Research Laboratory of Lawrence Berkeley National Laboratory specifically for energy policy analysis and emissions assessment at the provincial and municipal levels in China [20] featuring a good analysis of urban energy source distribution and consumption channels and dividing cities into separate modules for industrial sources, mobile sources, and nonenergy activities [20]. The design idea fits well with the implementation of environmental optimization policies by sector in PTCP. This study builds the structure of the Panzhihua LEAP model based on the GREAT tool, and the structure is shown in Figure 2. The Panzhihua LEAP model is divided into three main modules: industry, transport, and transmission, and on top of these three basic modules, there are several secondary and tertiary modules. The industrial framework includes separate modules for iron and steel, independent coking, construction (brick and tile, concrete), titanium (mainly in the titanium dioxide industry), and mining, with direct and indirect energy consumption, including heat, electricity, and additional facilities such as desulphurization, inserted into the subproduction lines. The transport framework covers mainly buses and trucks based on market flows such as the sale and retirement of vehicles, kilometers, and age. The transmission framework consists of power generation modules and energy losses. Electricity has certain losses in transmission and storage [21]. All fossil energy inputs are imported from the outer boundary.

### 2.2. Formulas

According to the IPCC’s (The Intergovernmental Panel on Climate Change) “Guidelines for National Greenhouse Gas Inventories” [22], the calculation of carbon emissions from energy consumption covers three leading greenhouse gases, including carbon dioxide, methane, and nitrous oxide. Each greenhouse gas contributes to global warming on a GWP basis, and this study uses a 100-year GWP scale to measure the contribution of greenhouse gases, and a value of 1 for carbon dioxide, 30 for methane, and 265 for nitrous oxide [22], with the calculated carbon emissions expressed in carbon dioxide equivalent.

**Figure 3:**
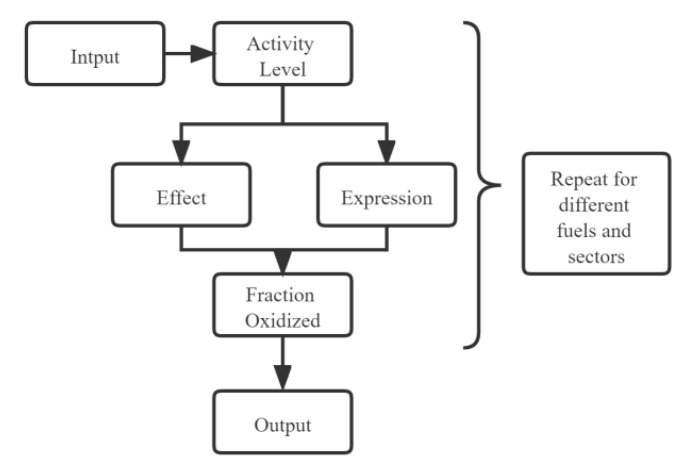
Application of formulations to the LEAP model (this figure was constructed for this study).

As shown in Figure 3, the GHG emissions calculations in the LEAP model use the IPCC method; these formulations were applied to constructing the Panzhihua LEAP model for this study (Equations (1) and (2)). In the LEAP model back-end program, the critical data were evaluated through “Effect”, “Expression”, “Fraction Oxidized”, and “Activity Level” modules and were output according to the following equation principles:

(1) $${\mathrm{GHG}}_{\mathrm{Fuel}}=\sum_{i}\sum_{j}AC_{i,j}\times NCV_{j}\times \left(EF_{{\mathrm{CO}}_{2}}+EF_{{\mathrm{CH}}_{4}}\times 30+EF_{N_{2}O}\times 265\right)$$

where ${\mathrm{GHG}}_{\mathrm{Fuel}}$ refers to the carbon emissions from energy consumption in the model, as an end result of the model, in tCO_2e_; AC refers to the activity level of the fuel, which is entered into the model via the “Activity Level” module in t; NCV refers to the calorific value of the fuel, as detailed in Table 1, which is stored in the LEAP model via the “Effect” module and is aligned with the IPCC database via the internet in KJ/kg or KJ/Nm^3^; EF refers to the carbon emission factor in kg/TJ, where the values of $EF_{CH_{4}}$ and $EF_{N_{2}O}$ for different fuels are detailed in Table 1; the calculation formula is as follows:

(2) EFCO2=CC×O×4412 

where $EF_{{\mathrm{CO}}_{2}}$ is the CO_2_ emission factor in kg/TJ; CC refers to the carbon content per unit calorific value, which is present in the LEAP model through the “Expression” module and corresponds to the “Effect” module above in tC/TJ (see Table 1 for details of different fuels); O is the oxidation rate, and it is entered in the model as the “Fraction Oxidized” module (see Table 1 for details of oxidation rates of different fuels); 44/12 is the conversion factor of carbon atoms to CO_2_ and the relative molecular mass, and it is the system’s default setting.

### 2.3. Scenario

Panzhihua City is located at the junction of Sichuan and Yunnan Province, with a partially integrated industrial structure and many intensive chemical enterprises [6]. In 2019, the coal consumption of industrial enterprises above the scale in Panzhihua was 8,879,000, accounting for 14% of the total coal consumption in the province, much greater than that of other cities, of which the coal consumption of three industries—coking, iron and steel, and construction materials—accounted for more than 55% of the total coal consumption of the regulated industries, respectively [23]. In order to reduce the environmental pressure of air pollutant emissions from industrial and mobile sources, the PTCP’s key strategy focuses on the remediation of boilers in enterprises around urban areas, end-of-pipe treatment of critical industries, and control of heavy vehicles. The PTCP states that the proposed control of these three areas will lead to a sustained reduction in the environmental pressure of air pollution. Panzhihua City has proposed three years, from 2022 to 2024, for the profound transformation of pollutant management in iron and steel, construction materials (brick and tile, concrete), titanium powder, coking, and mining in Panzhihua City in terms of the PTCP strategy. There has been a complete banning of coal-fired and gas-fired boilers of 35 steam tons and below in the built-up area of the city, and the complete banning of coal-fired and gas-fired boilers of 10 steam tons and below in other areas, all of which will be replaced by electricity-fired boilers by the end of 2023 [8,9]. The PTCP targets enterprises above the scale for desulphurization, denitrification, dust removal, and VOCs removal, accurate to each production line or kiln, and the quantity statistics are shown in Table 2.

In 2019, Panzhihua City had 293,000 vehicles, including 0.7 million heavy-duty vehicles, emitting 0.27 million tons of NO_X_ annually, accounting for 54% of total vehicle emissions [8]. Panzhihua City has implemented a replacement policy for the number of internal combustion engines (ICE), and by the end of 2023, it will have completely phased out China III and below diesel trucks; by the end of 2024, it will have further phased out 10% of the city’s China IV diesel trucks on top of the complete phase-out of China III and below diesel trucks. Regarding the acceleration of the promotion of new energy vehicles (NEA): by the end of 2022, 50% of buses and 10% of trucks in the city will be renewed with new energy vehicles, while the proportion of new energy vehicles among newly purchased vehicles will reach 10%; by the end of 2023, 80% of buses and 20% of trucks in the city will be renewed with NEA vehicles, while the proportion of NEA vehicles among newly purchased vehicles will reach 15%; by the end of 2024, all buses and 30% of trucks in the city will be replaced with NEA vehicles, and 20% of the new vehicles will be NEA vehicles [8,9].

The PTCP is based on 2019 data. It aims to improve air quality by 2022–2024, decomposing the city’s overall targets into districts, counties, and points and deploying actionable and targeted subannual attack measures for industrial enterprises and the transport sector, taking into account the actual situation in the city [8,9]. Therefore, this study took 2019 as the base year. By the scope delineated in the above-mentioned three-year plan, the Panzhihua LEAP model was specified to take key industrial enterprises (iron and steel, coking, building materials, titanium dioxide, mining) and the heavy vehicle component of road traffic (buses and trucks) as the system boundary, and to build an energy conversion component in conjunction with the provincial grid structure. Four simulation scenarios were established to assess the carbon emissions of Panzhihua City from 2020 to 2024.

#### 2.3.1. Baseline Scenario

The base-year scenario is the “zero scenario”, in which the vehicle type and energy use structure of enterprises were maintained at the 2019 level, no environmental control emission measures were taken until 2024, and only normal enterprise consumption and natural growth of motor vehicles were modelled.

#### 2.3.2. Boiler Remediation (BR)

The boiler remediation scenario added to the baseline scenario, the second scenario of 12 industrial enterprises replacing boilers under 35t/h with electric boilers by the end of 2023, and 2 industrial enterprises replacing boilers under 35t/h with electric boilers by the end of 2024.

#### 2.3.3. Pollutant Treatment Upgrade (PTU)

The pollutant treatment upgrade scenario added the calculation modules for iron and steel, independent coking, construction (brick and tile, concrete), titanium, and mining to the baseline scenario. The scenario was built for 44 production lines of iron and steel enterprises, 5 production lines of independent coking enterprises, 27 of construction enterprises, 21 of titanium enterprises, and 9 of mining enterprises. Secondary scenarios such as desulphurization, denitrification, VOCs (volatile organic compounds) removal, and dust removal treatment were covered.

#### 2.3.4. Traffic Improvement (TI)

The traffic improvement scenario added a market module for the city’s sale and retirement of buses and trucks to the baseline scenario. It intervened in the free market following policy take-up. The scenario included a second scenario with alternating new energy and conventional fuels and modeled carbon emissions under changing vehicle age and kilometers.

## 3. Results and Discussion

This study built the Panzhihua LEAP model based on the 2019 industrial enterprise activity-level data and vehicle ownership in Panzhihua City from the Sichuan Provincial Air Quality Regulation and Control Integrated Decision Support Platform [24], combined with one iron and steel enterprise above the scale, one independent coking, twenty-seven construction material enterprises, twenty-one titanium dioxide enterprises and nine mining enterprises incorporated in the PTCP. In the power structure of Panzhihua City, the proportion of hydropower can reach 91.6% and above in the abundant water period, and in addition to hydropower, Panzhihua City also has wind power generation, whose total installed capacity is over 1840 MW [25]. Local electricity in the city and state is given priority to enter the provincial grid before being distributed to the regions, and according to the Sichuan Yearbook Statistics 2020, it is known that clean energy in Sichuan province accounted for about 85% of 2019 [26].

**Figure 4:**
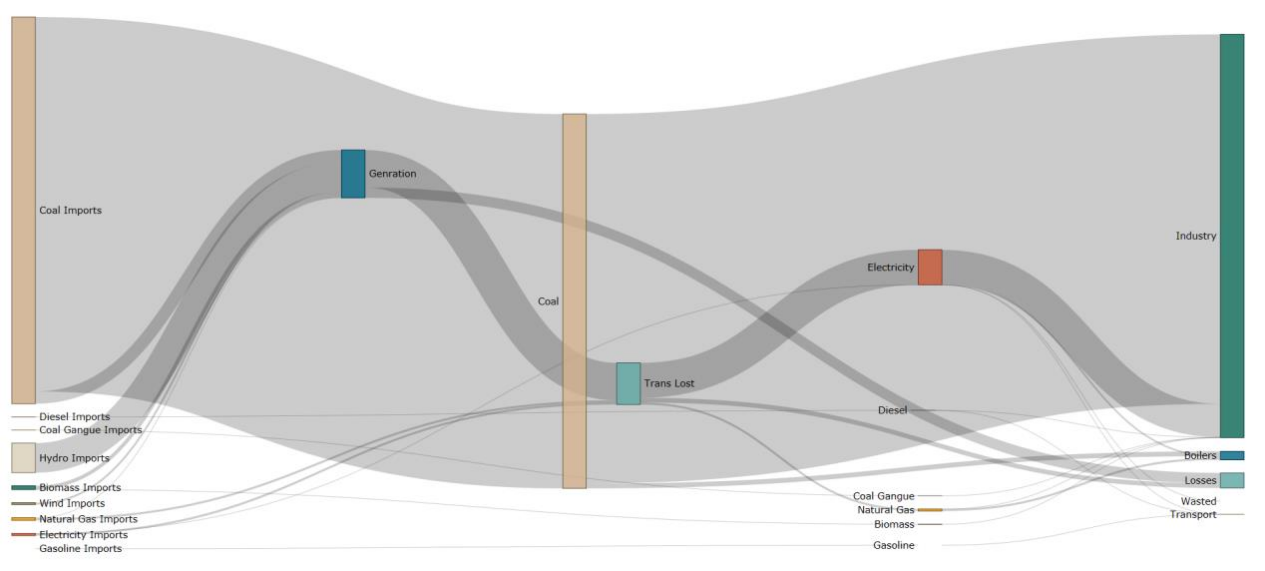
The Sankey Diagram of the Panzhihua LEAP model (this figure was constructed for this study).

The Sankey Diagram of the Panzhihua LEAP model is shown in Figure 4. The LEAP model simulation analysis shows that the energy for the industrial and road transport components of Panzhihua City comes from hydroelectric power generation, thermal power generation, biomass power generation, wind power generation, coal-based fuels (including bituminous and anthracite coal, etc.), natural gas, diesel, gasoline, biomass, and coal gangue transferred in from the system boundary, respectively. According to Table 3, the structure reveals an energy loss of approximately 12.7% in the transmission and storage of electricity production. The largest share of industrial energy consumption is 115.4 million GJ, representing about 88.7% of the total demand.

The carbon emissions generated by the various sectors in Panzhihua in the base year are shown in Table 4. In the 2019 base year, Panzihua City generated 9936.83 thousand metric tonnes CO_2_ equivalent carbon emissions overall based on the scope of the PTCP policy envelope. The carbon emissions results are similar to the energy balance, with the industrial sector generating the most significant carbon emissions of 9775.63 thousand metric tonnes of CO_2_ equivalent, accounting for 98.38% of the total carbon emissions. The boiler sector (boilers are counted separately and are not included in the industrial result) generated 161.2 thousand metric tonnes of CO_2_ equivalent of carbon emissions, accounting for only 1.62% of the total. The transport component, consisting of buses and vans, generated 95 metric tonnes of CO_2_ equivalent, a small contribution to the city’s overall carbon emissions. Amongst the industrial sector, the iron and steel sector is particularly prominent, contributing 89.93% of the total industrial emissions.

### 3.1. Urban Power Generation

**Figure 5:**
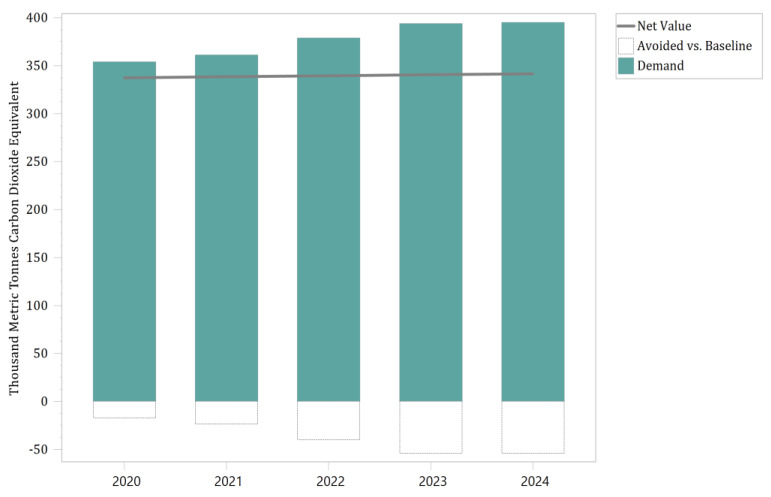
Carbon emissions from urban electricity generation under simulation versus base year.

The power generation module of the LEAP model produced a different carbon intensity than the baseline scenario when the BR, PTU, and TI scenarios were superimposed. As shown in Figure 5, the carbon intensity of electricity in Panzhihua City increased yearly, and the net value showed a smoothly rising curve. In contrast, the value of the carbon demand required for electricity generation was always higher than the net value. The difference between the carbon intensity of electricity in Panzhihua in the three simulations and the carbon intensity of electricity in the baseline scenario is significant, and the distance between the two increases over time.

As shown in Table 5, from 2020 to 2024, the difference between the carbon intensity of electricity in the three simulations and that of the baseline scenario increased from 16.58 thousand metric tonnes of CO_2_ equivalent to 53.6 thousand metric tonnes CO_2_ equivalent, an increase of 2.23 times in four years, or an average annual increase of 36.5%. The enhancement of the data strongly suggests that the BR, PTU, and TI scenarios lead to a growth in electricity load and drive an increase in carbon emissions’ intensity. The overall five-year intensity of urban electricity demand is 1883.8 thousand metric tonnes CO_2_ equivalent, rising from 354.03 thousand metric tonnes CO_2_ equivalent to 395.24 thousand metric tonnes CO_2_ equivalent by year, an estimated increase of 11.6%. The overall carbon intensity in the baseline scenario is 1697.68 thousand metric tonnes of CO_2_ equivalent, which is 186.12 thousand metric tonnes of CO_2_ equivalent below the carbon emissions of the three scenarios above. By the end of the simulation, the base scenario improved from 337.45 thousand metric tonnes of CO_2_ equivalent to 341.64 thousand metric tonnes of CO_2_ equivalent, an increase of about 1.2%. The variation in the magnitude of the curve rise indicates that the pressure on power carbon intensity has a more aggressive expansion in the BR, PTU, and TI scenarios.

### 3.2. Boilers

The boiler remediation scenario is a modular model explicitly built for 35 t/h steam-tonne boilers. Fourteen industrial enterprises in Panzhihua City will replace part of their boilers with electric boilers by 2024. Electric boilers have a higher energy consumption than conventional boilers, without any reduction in output, and the energy consumption of different fuels (including electricity) is uniformly converted into gigajoules of heat in the LEAP model, in which case the energy demand is more intuitively shown, and also validates the above statement. As shown in Table 6, the overall model demand was 124.98 thousand gigajoules of heat over five years, with the total energy consumption of electricity being much greater than that of the other fuel types (coal refers to the aggregate of coal-based fuels, including anthracite and bituminous coal, but excluding gangue). By 2023 and 2024, when almost all boilers in the module will be replaced with electric boilers, the module’s energy demand for coal and natural gas is already negligible. The total energy consumption reached 2533.67 thousand gigajoules in 2024, up from 2434.81 thousand gigajoules in 2020, an increase of about 4%, in which the electricity consumption increased about 1.46 times.

**Figure 6:**
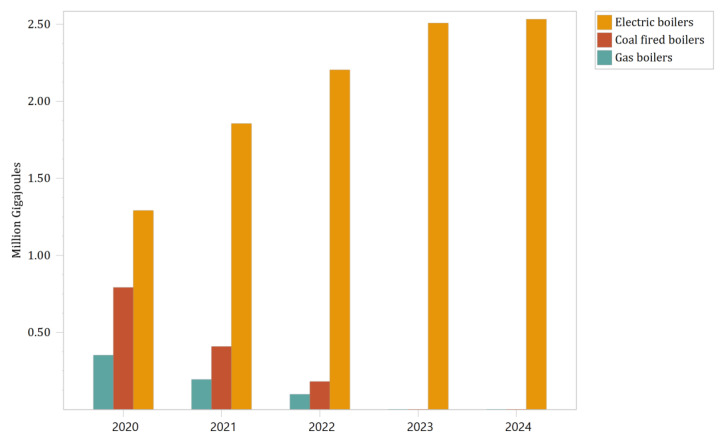
Boiler module energy demand trend in the boiler remediation scenario.

In Figure 6, the energy mix of boilers changed under the policy intervention, which in general was a significant decline in the demand for energy other than electricity and a gradual increase in the demand for electricity. In 2021, coal and natural gas decreased by 33% year-on-year. Electricity increased by 47% year-on-year. In 2022, coal and natural gas decreased by 50% versus 49% year-on-year. In 2023, electricity increased by 33% year-on-year. Coal and natural gas demand will be 0 in 2024. After 2022, Electricity growth showed a weakening of approximately 23.1% to 30.6% per year. The reason for this is that companies are constrained by capacity constraints such as raw materials and the need to ensure that the total calorific value of new boilers remains stable in 2023 in anticipation of the complete removal of conventional boilers, creating a sharp and then slow demand trend for electricity’s calorific value.

**Figure 7:**
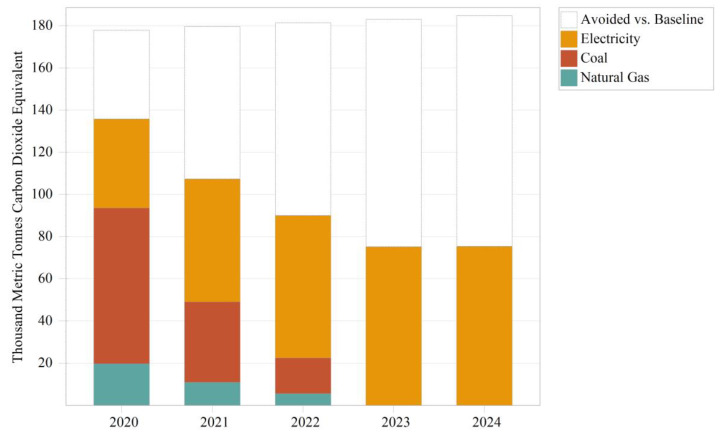
Carbon emissions (direct and indirect carbon) for the boiler module under the boiler remediation scenario and the difference plotted against the baseline scenario.

The carbon emissions predicted by the boiler module in the Panzhihua LEAP model in the boiler remediation scenario are shown in Figure 7, where the white part indicates the difference between the baseline scenario and the boiler remediation. From the Panzhihua Yearbook Statistics 2020 [24], it shows that the enterprise is in a state of energy value-added by increasing the output value by 1% year by year under the phase of keeping relatively constant on the calorific value demand. It can be seen that the boiler in the coal-to-electricity scenario both ensures the calorific capacity and allows carbon emissions to be reduced year by year and is much smaller than the carbon emissions in the base year, with a significant carbon reduction effect.

As seen in the detailed data in Table 7, the boiler remediation scenario reduces total carbon emissions by 375.45 thousand metric tonnes of CO_2_ equivalent over the five-year period compared to the baseline scenario, with the most significant improvement between 2020 and 2021, where the reduction in emissions in 2021 is 0.97 times the reduction in the previous year. Although the improvement in carbon emissions is significant year-on-year, the relative increase decreases, from 97% in the model’s second year to 36% in 2023, before leveling off at 1.4% in 2024. This increase is consistent with the trend in energy demand, which shows that coal and natural gas combustion account for a large share of the contribution to carbon emissions. As Panzhihua City’s electricity structure has large-scale clean sources, such as hydropower and wind power, the carbon reduction effect of replacing traditional energy sources with electricity at a specific calorific value is noticeable. Therefore, the boiler remediation scenario is promoted in Panzhihua City not only for general environmental management, but also for carbon emission control; it is a synergistic measure to reduce pollution and carbon emissions.

### 3.3. Industry

**Figure 8:**
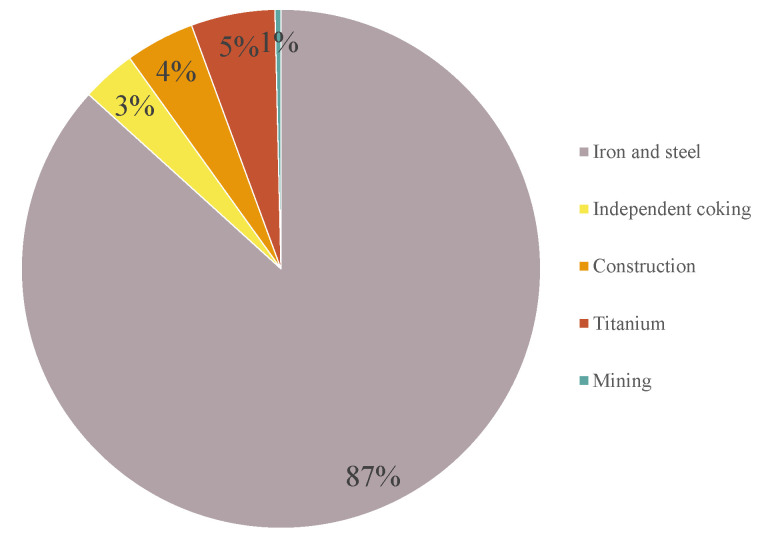
Energy demand share of industries in Panzhihua City.

The pollutant treatment upgrade scenario is a differentiated scenario that adds the use of desulphurization and dust removal facilities, etc., to the baseline scenario. In the LEAP model, the corresponding end-of-pipe treatment facility modules are installed for the different production lines of the 63 industrial enterprises, and the corresponding scenario endpoints are set up in the model according to the target years required in the three-year plan. The iron and steel enterprises in Panzhihua City are long-process. Compared to short-process, it combines many front-end raw material preparation processes such as lime kilns, coking, sintering, and pellet plants and has a certain number of LF and RH refiners and converters [27]. As a result, Panzhihua iron and steel is responsible for a significant energy contribution to the city as a whole. As shown in Figure 8, the steel enterprise module in the Panzhihua LEAP model requires 87% of the overall energy, with the remaining 5% for titanium, 4% for construction, 3% for independent coking, and 1% for mining, in that order. Of these, in the construction enterprise module, concrete accounts for another 98.5%, and brick and tile only 1.5%.

The industrial module increases by about 1% per year according to the industry capacity projections in the Panzhihua Yearbook Statistics 2020 [24]. As shown in Table 8, the priority industrial system modules required 592.83 million gigajoules of energy in the pollutant treatment upgrade scenario. From 2020 to 2024, the overall energy demand progressively increased by between 1% and 1.91%, with a peak in 2022. As can be seen from Table 2 above, the renovation of the vital industry modules is concentrated in 2022 and 2023 (desulphurization and denitrification as well as dust removal), with a subtotal of 80 additions or upgrades of treatment facilities in steel companies, 47 in construction materials, 9 in mining, and 10 in independent coking at the end of the five-year simulation, for a total of 146 additions or upgrades, with a ratio of energy consumption to the number of renovations of approximately 4.23 × 10^4^ gigajoules per item. As the overall energy consumption of the industry (direct and indirect consumption of fuel) is greater than the energy consumed by the treatment facilities, the change in energy consumption brought about by the treatment facilities is less significant.

**Figure 9:**
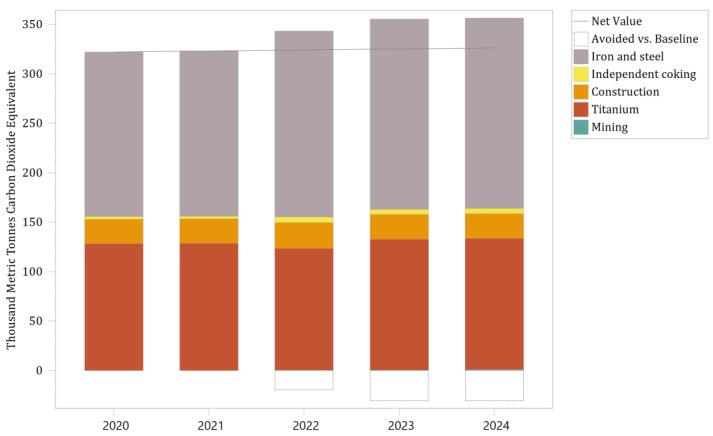
Indirect carbon emissions from key industrial modules under the pollutant treatment upgrade scenario and the difference plotted against the baseline scenario.

The indirect carbon emissions predicted for the pollutant treatment upgrade scenario for the critical industrial module in the Panzhihua LEAP model are shown in Figure 9 and Table 9. The white areas in Figure 9 represent the difference from the baseline scenario. The model presents the results using indirect carbon emissions, as the overall energy consumption of the critical industrial module is relatively strong. Indirect carbon emissions refer to carbon emissions from end-use consumption through nondirect combustion energy sources, such as electricity input, heat input, etc. [28,29]. The overall carbon emissions are correspondingly high, and the change in carbon emissions brought about by the treatment facilities is insignificant. The key sectors’ most significant contributor to carbon emissions is the iron and steel industry, followed by titanium dioxide, construction materials, independent coking, and mining. The carbon emissions contribution of these sectors is primarily in line with their share of energy demand. It is worth noting that titanium dioxide, construction materials, independent coking, and mining account for 5%, 4%, 3%, and 1% of energy demand, but their contribution to carbon emissions was approximately 37%, 7%, 1%, and 0.08%. The difference between titanium dioxide and construction materials in these two comparisons is significant, and the reasons for this difference depend mainly on the industry’s process and feedstock profile. The raw materials used for titanium dioxide are titanium concentrate, rutile, and titanium slag. Excluding coal consumption, which provides the calorific value, these raw and auxiliary materials tend to generate additional carbon emissions during the process [30]. Many enterprises in the construction materials industry use fuels other than coal, such as gangue and biomass. As mentioned in Table 1, the calorific value of these fuels is lower, and the carbon content is almost equivalent to that of coal. Compared to coal, if these fuels need to reach the required calorific value, the kiln will need to consume more quickly, resulting in additional carbon emissions.

As a result of the intensive construction and commissioning of the treatment facilities, the carbon emissions of the priority industry module significantly exceed the baseline scenario from 2022 to 2024, with negative growth in carbon reduction effectiveness. Up to the end of the simulation, the focus industry module generated indirect carbon emissions of 1700.58 thousand metric tonnes CO_2_ equivalent, which exceeded the baseline scenario of 79.66 thousand metric tonnes CO_2_ equivalent. In terms of growth, indirect carbon emissions are increasing every year. The average annual increase in carbon emissions under the pollutant treatment upgrade scenario was about 2.1% compared to the previous year’s emissions, which exceeded the average increase of 0.3% in the baseline scenario. The increase in the pollutant treatment upgrade scenario was about seven times greater, with a clear trend of carbon increase. In terms of the number of increases, there was an increase of approximately 34.28 thousand metric tons of CO_2_ equivalent in 2024 compared to 2020. Combined with the addition or upgrade of 146 governance facilities, the ratio of indirect carbon emissions to the number of retrofits was approximately 234.7 metric tons of CO_2_ equivalent per item. Although Panzhihua City’s electricity structure is relatively clean, indirect carbon emissions will be significantly higher for upgrades and new construction of desulphurization, denitrification, VOCs removal, and dust removal equipment that does not change the energy use structure and does not reduce energy consumption but instead increases fan airflow and increases combustion temperature. This is inconsistent with the PTCP’s desire to reduce environmental pressures through the end-of-pipe treatment of industrial enterprises. The additional treatment facilities may reduce atmospheric pollutants such as sulfur dioxide, nitrogen oxides, and particulate matter, but carbon emissions are elevated by increased energy consumption. Therefore, the promotion of end-of-pipe treatment in Panzhihua City, although it can improve air quality [8], will cause an increase in carbon emissions and is not a synergistic measure to reduce pollution and carbon emissions.

### 3.4. Road Traffic

**Figure 10:**
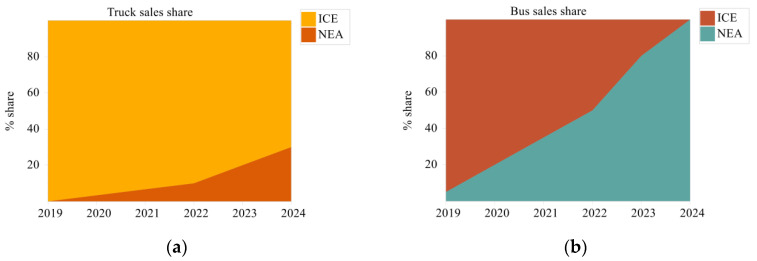

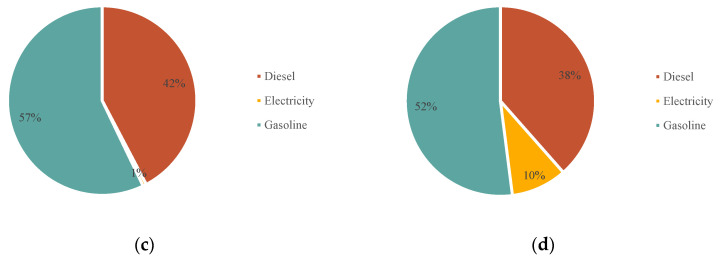
Sales and stock share of carriers in the traffic improvement scenario; (**a**) sales share of trucks; (**b**) sales share of buses; (**c**) stock share of the three energy vehicles in 2019; (**d**) stock share of the three energy vehicles in 2024).

The traffic improvement scenario is a differentiated scenario that adds new energy vehicles (NEA) to the baseline scenario for replacing internal combustion engine vehicles (ICE). From the Panzhihua Yearbook Statistics 2020 [24], it is known that the total stock of buses and trucks in the market in 2019 was 41.417 thousand units, and according to the motor vehicle market turnover in the ten years from 2010 to 2019, an average of 2.4 thousand buses and trucks were traded each year. In the Panzhihua LEAP transport module, the academic life of the vehicle is set at 22 years, a vehicle survival curve is added [31], and the free market for vehicles is interfered with by the policy requirements of the three-year plan. As shown in Figure 10, the market share of ICE and NEA vehicles for trucks and buses and the share of the stock of vehicles using different energy sources change under the traffic optimization scenario in the Panzhihua LEAP transport module. From 2019 to 2024, the proportion of NEA-type vehicles sold in vans and buses gradually increases, reaching a share of 30% and 100%, respectively, by 2024, while the market turnover of ICE-type vehicles for vans decreases to 70% and ICE-type vehicles for buses are no longer sold. As a result of policy interventions in market turnover, the share of the market stock of vehicles using electricity as an energy source is increasing from 2019 to 2024, from 1% to 10%, and the share of the market stock of vehicles using petrol and diesel is decreasing, from 57% and 42% to 52% and 38%, respectively.

**Figure 11:**
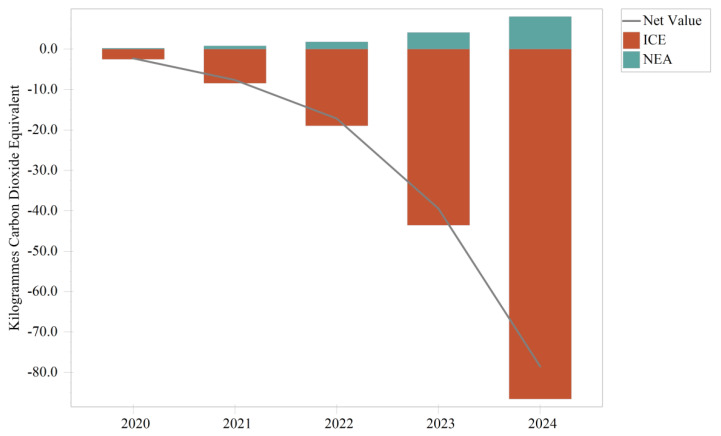
Carbon emissions of the transport module in the traffic improvement scenario versus the baseline scenario.

Figure 11 illustrates the difference between the two scenarios of carbon emissions from the transport module, i.e., the traffic improvement scenario compared to the baseline scenario. Positive values in the figure indicate that the portion of carbon emissions is more significant in the traffic improvement scenario than in the baseline scenario and vice versa for the reduced portion. Between 2020 and 2024, carbon emissions from the NEA category of vehicles in the traffic improvement scenario were higher than in the base case, increasing by an average of 1.96 kg CO_2_ equivalent per year until 2024, when they exceeded the base case by 8.09 kg CO_2_ equivalent. In contrast, carbon emissions from ICE vehicles were below the baseline scenario each year, decreasing by an average of 31.5 kg CO_2_ equivalent per year until 2024, when they were below the baseline scenario of 86.59 kg CO_2_ equivalent. Due to the significant negative carbon contribution of ICE vehicles in the traffic improvement scenario, the difference in overall carbon reduction compared to the positive carbon contribution of NEA was over 9.46 times greater with the replacement of ICE by NEA vehicles.

As shown in Table 10, the transport module emitted a total of 4152.38 kg CO_2_ equivalent over the five years in the traffic improvement scenario, compared to a total reduction of 144.92 kg CO_2_ equivalent in the baseline scenario. The emissions of ICE vehicles decreased from 890.68 kg CO_2_ equivalent to 784.47 kg CO_2_ equivalent, an average annual reduction of 3.1%. The NEA vehicles benefit from Panzhihua City’s clean electricity to keep their carbon emissions consistently reduced. Therefore, implementing the transport optimization policy in Panzhihua City can reduce the pressure of air pollution [8] and reduce the carbon emissions of road traffic, so it is a pollution and carbon reduction measure.

### 3.5. Overall Effectiveness

**Figure 12:**
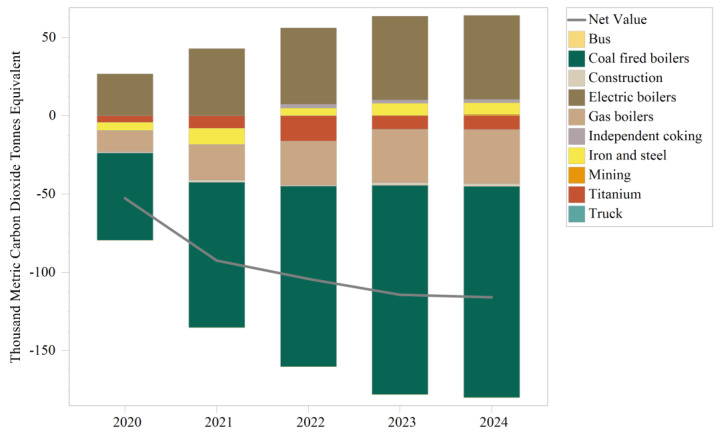
Plot of total carbon emissions for each module against the baseline scenario.

The LEAP model was analyzed in a five-year simulation using the GREAT tool in combination with three demand scenarios such as boiler remediation, pollutant treatment upgrade, and traffic improvement, and the total value of carbon emissions from each module was aggregated to produce the results in Table 11 and Table 12 and Figure 12. As shown in Table 8, the Panzhihua LEAP model predicts that crucial industries and heavy vehicles in Panzhihua City will generate 52.41 million metric tons of CO_2_ equivalent carbon emissions over five years. The significant contribution from the key industries will be 51.95 million metric tonnes CO_2_ equivalent, the minor contribution from boilers will be 0.47 million metric tonnes CO_2_ equivalent, and the minor contribution from the traffic component will be 4.14 × 10^−6^ million metric tonnes CO_2_ equivalent. Overall, carbon emissions are increasing yearly at an average rate of 0.8%.

Figure 12 shows the difference between the total carbon emissions of each module in the three-in-one scenario and the baseline scenario, with positive values indicating more significant carbon emissions than in the baseline scenario and negative values indicating weaker carbon emissions than in the baseline scenario. The net value in the graph showed negative growth, indicating that the overall carbon emissions in the three-in-one scenario decreased year by year compared to the baseline scenario. The replacement of coal-fired and gas-fired boilers provided the most considerable emission reduction contribution to the system. Since some of the titanium dioxide and building materials enterprises are part of the coal-fired boilers described in the conversion to electricity, these two components produce harmful emissions in the three-in-one scenario, which are objectively lower than the carbon emissions in the baseline scenario. The remaining sectors that contributed to emissions reductions were trucks and buses. However, they were all negative contributors. In descending order, they are: electric boilers, independent coking, iron and steel, and mining. Table 12 shows that the Panzhihua LEAP system would reduce overall carbon over the five years from 2020 to 2024 in the three-in-one scenario. Over the five years, the overall cumulative carbon reduction is 479.37 thousand metric tonnes CO_2_ equivalent, with a maximum reduction of 115.82 thousand metric tonnes CO_2_ equivalent in 2024. The carbon reduction in 2024 compared to 2020 (52.67 thousand metric tonnes CO_2_ equivalent) is characterized by a factor of 1.19. By sector, most sectors with a negative contribution to carbon reduction shift from a positive to a negative contribution from 2023, such as mining, iron and steel, independent coking, and electric boiler sectors. This is due to the explicit requirement for end-of-pipe treatment facilities in the industrial sector to be built and operational by 2023–2024, as well as the requirement to replace all boilers in the coal-to-electric boiler scenario by 2023–2024. Most sectors that contributed significantly to the reduction in 2021 were coal-fired boilers, gas-fired boilers, titanium dioxide, construction materials, and traffic, as they all started to withdraw most of their fossil energy-using facilities in 2021 in order to meet the standards in 2023.

## 4. Conclusions

In this study, the LEAP model for Panzhihua City was developed through the GREAT tool, and the carbon emission structure and specific emissions of Panzhihua City for the five-year period from 2020 to 2024 under the new policies were simulated and analyzed through four major scenarios (boiler remediation, pollutant treatment upgrade, traffic improvement, and baseline scenario). The results of the Panzhihua LEAP model show that the overall carbon emissions of Panzhihua City would decrease by 479.37 thousand metric tons of CO_2_ equivalent if all policies were implemented simultaneously. The boiler remediation scenario will contribute positively to the carbon reduction under the different scenarios, and the model will reduce carbon emissions by 539.87 thousand metric tonnes of CO_2_ equivalent. The pollution abatement scenario would negatively contribute, and the model would increase carbon by 76.21 thousand metric tons CO_2_ equivalent. The transport optimization scenario would positively contribute to and reduce carbon by 0.15 thousand metric tons CO_2_ equivalent. From the data results, it is clear that the implementation of coal-to-electricity conversion measures for boilers under 35t/h steam tonnage is a beneficial reduction in carbon emissions when implementing general air quality assurance measures in cities with relatively clean grid structures. Regarding Panzhihua City, carbon reductions of over 70.5% can be achieved in five years. At the same time, introducing new energy vehicles to replace heavy vehicles in the city can also effectively reduce carbon emissions. However, the degree of reduction depends on the depth of the measures to market intervention. For example, Panzhihua City, in the provision of heavy vehicles in the market intervention, still left about 68.34% of a large number of non-new energy vehicles in the market, and carbon emissions in five years saw only a 3.4% reduction. Furthermore, although end-of-pipe treatment is a standard measure to combat air pollution in the industrial sector, it is not beneficial in terms of reducing carbon emissions, as shown by the simulation results of Panzhihua LEAP model, as large equipment such as desulphurization, denitrification, VOCs removal facilities, and dust removal facilities do not intervene in production source consumption, as it is independent energy-consuming equipment added to the external boundaries of the system, increasing the overall energy consumption of production. It also increases carbon emissions. Considering the impact of carbon reduction, environmental measures such as source replacement or energy efficiency should be promoted in developing environmental protection measures. Our results have pointed out a clear direction for reducing carbon emissions and paved the way for more detailed studies of specific protocols. In future research, the carbon footprint of the city as a whole can be studied in more detail to discuss the impact of industrial pollutant reduction measures and road motor vehicle management on carbon reduction. Quantitative analysis can be carried out to identify measures with carbon reduction benefits, such as converting coal boilers to electricity and promoting new energy vehicles.

## Figures and Tables

**Table 1:** Calculation parameters for carbon emissions from energy consumption.

Fuel Type		Calorific Value	Carbon Content	Oxidation Rate	CH_4_ Emission Factors	N_2_O Emission Factors
		KJ/kg or KJ/Nm^3^	tC/TJ	%	kg/TJ	kg/TJ
Solids	Coal bituminous	23,204	25.8	93	10	1.4
	Cleaned Coal	26,344	25.4	93	10	1.5
	Coal Gangue	3349	26.4	92	10	1.4
	Biomass	14,650	29.9	90	30	4
	Diesel	43,330	20.2	98	5	0.6
	Gasoline	44,800	18.9	98	20	0.6
Gases	Natural Gas	38,931	15.3	99	5	0.1
	Coke oven gas	17,385.4	13.6	99	1	0.1
	Blast furnace gas	3769	70.8	99	1	0.1

Data based on Guidelines for National Greenhouse Gas Inventories [22]; general rules for calculation of the comprehensive energy consumption (GB/T 2589-2020) [23].

**Table 2:** Statistics on the number of key industrial pollutant treatment facilities renovated.

Pollutant Treatment Measures	Industries Involved and Corresponding Target Years								
	Iron and Steel		Construction			Mining		Independent Coking	
	2022	2023	2022	2023	2024	2023	2024	2022	2023
Additional denitrification	17	2	-	-	-	-	-	1	3
Denitrification upgrade	2	2	2	-	-	-	-	-	-
Additional desulphurization	20	3	-	19	2	-	-	1	2
Desulphurization upgrade	7	2	-	-	-	-	-	-	-
Additional dust removal	5	1	-	6	1	-	-	-	-
Dust removal upgrade	7	12	17	-	-	3	6	3	-
VOCs removal	-	-	-	-	-	-	-	1	-

The counts in the table are an aggregation of the number of equipment that needs to be retrofitted in key industries across the city.

**Table 3:** Panzhihua LEAP model energy balance sheet (base year).

System Boundaries	Solid Fuels	Natural Gas	Hydropower	Renewables	Biomass	Electricity	Oil Products	Total
Imports	110,684.65	771.38	8467	546.25	1069.25	607.46	19.86	122,165.87
Generation	−3505.15	−166.9	−8467	−546.25	−1015.69	10,724.2	-	−2976.82
Trans Lost	-	−11.48	-	-	-	−1314.47	-	−1325.95
Industry	105,789.94	0.59	-	-	53.552	9564.32	19.85	115,428.27
Transport	-	-	-	-	-	0.00003	0.012	0.012
Boilers	1389.54	592.38	-	-	-	452.87456	-	2434.80

Units: thousand gigajoule.

**Table 4:** Carbon emission values by sector in Panzhihua (Base Year).

Branch		Value	Total
Boilers	Coal fired boilers	128.38	161.2
	Gas boilers	32.82	
	Electric boilers	-	
Industry	Construction	387.10	9775.63
	Independent coking	351.62	
	Iron and steel	8791.51	
	Mining	39.52	
	Titanium	205.87	
Transport	Bus	2.53 × 10^−5^	9.50 × 10^−4^
	Truck	9.25 × 10^−4^	
Total			9936.83

Units: thousand metric tonnes CO_2_ equivalent.

**Table 5:** Table of carbon emissions from urban electricity generation under the simulation compared to the base year.

Branch	2020	2021	2022	2023	2024	Total
Avoided vs. Baseline	−16.58	−22.96	−39.53	−53.44	−53.60	−186.12
Baseline	337.45	338.48	339.53	340.58	341.64	1697.68
City Demand	354.03	361.45	379.05	394.02	395.24	1883.80

Units: thousand metric tonnes CO_2_ equivalent.

**Table 6:** Table of boiler module energy requirements in the boiler remediation scenario.

Branch	2020	2021	2022	2023	2024	Total
Electricity	1027.43	1511.52	2005.19	2508.59	2533.67	9586.40
Coal	1042.16	701.72	354.37	-	-	2098.25
Natural Gas	365.22	245.92	124.19	-	-	735.32
Total	2434.81	2459.16	2483.75	2508.59	2533.67	12,419.98

Units: Thousand Gigajoules.

**Table 7:** Table of boiler module carbon emissions between BR and baseline scenarios.

Branch	2020	2021	2022	2023	2024	Total
Total of Baseline	178.07	179.75	181.44	183.15	184.87	907.27
Electricity	34.03	48.34	62.02	75.16	75.33	294.87
Coal	97.25	65.48	33.07	0.00	0.00	195.80
Natural Gas	20.44	13.76	6.95	0.00	0.00	41.15
Avoided vs. Baseline	26.35	52.16	79.40	107.99	109.55	375.45

Units: thousand metric tonnes CO_2_ equivalent.

**Table 8:** Table of energy demand for key industries under pollutant treatment upgrade scenarios.

Branch	2020	2021	2022	2023	2024	Total
Iron and steel	100.10	101.10	103.00	104.43	105.48	514.11
Independent coking	3.89	3.93	4.06	4.10	4.14	20.11
Construction	4.98	5.03	5.16	5.21	5.26	25.63
Titanium	6.04	6.10	6.16	6.22	6.28	30.80
Mining	0.43	0.43	0.44	0.44	0.45	2.18
Total	115.43	116.58	118.81	120.40	121.61	592.83

Units: million gigajoules.

**Table 9:** Indirect carbon emissions from key industrial modules under the pollutant treatment upgrade treatment scenario and differences from the baseline scenario.

Branch.	2020	2021	2022	2023	2024	Total
Total of Baseline	322.19	323.18	324.18	325.18	326.20	1620.93
Iron and steel	166.56	167.07	188.19	192.31	192.53	906.67
Independent coking	2.76	2.77	5.57	5.33	5.34	21.77
Construction	24.54	24.62	26.25	25.12	25.15	125.68
Titanium	128.32	128.72	123.31	132.30	132.45	645.09
Mining	0.01	0.01	0.01	0.34	1.02	1.39
Avoided vs. Baseline	0.00	0.00	−19.15	−30.23	−30.28	−79.66

Units: thousand metric tonnes CO_2_ equivalent.

**Table 10:** Table of differences between the carbon emissions of the transport module in the traffic improvement scenario and the baseline scenario.

Branch	2020	2021	2022	2023	2024	Total
Total of Baseline	894.28	854.83	835.95	840.56	871.69	4297.30
ICE	890.68	845.45	816.16	796.29	784.47	4133.05
NEA	1.35	1.77	2.65	4.85	8.72	19.33
Avoided vs. Baseline	2.25	7.61	17.13	39.43	78.50	144.92

Units: kilograms CO_2_ equivalent.

**Table 11:** Table of total carbon emissions under simulation.

Branch	2020	2021	2022	2023	2024	Total
Industry	10.19	10.28	10.39	10.5	10.6	51.95
Transport	8.92 × 10^−7^	8.47 × 10^−7^	8.18 × 10^−7^	8.0 × 10^−7^	7.91 × 10^−7^	4.14 × 10^−6^
Boilers	0.14	0.11	0.09	0.07	0.07	0.47
Total	10.32	10.38	10.47	10.57	10.67	52.41

Units: million metric tonnes CO_2_ equivalent.

**Table 12:** Table of total carbon emissions for each module versus the baseline scenario.

Branch	2020	2021	2022	2023	2024	Total
Electric boilers	26.86	42.94	48.80	53.60	53.66	225.86
Independent coking	−0.09	−0.17	2.34	2.09	2.09	6.26
Iron and steel	−5.31	−10.33	5.02	7.76	7.51	4.65
Mining	−2.97 × 10^−4^	−5.77 × 10^−4^	−1.16 × 10^−3^	0.30	0.92	1.22
Bus	−3.36 × 10^−7^	−1.08 × 10^−6^	−2.43 × 10^−6^	−5.05 × 10^−6^	−8.92 × 10^−6^	−1.78 × 10^−5^
Truck	−1.95 × 10^−6^	−6.63 × 10^−6^	−1.5 × 10^−6^	−3.51 × 10^−5^	−7.10 × 10^−5^	−1.29 × 10^−4^
Construction	−0.78	−1.52	−0.62	−1.79	−1.83	−6.55
Titanium	−4.09	−7.96	−16.01	−8.53	−8.74	−45.34
Gas boilers	−13.47	−22.59	−28.32	−34.15	−34.49	−133.03
Coal fired boilers	−55.78	−92.80	−115.35	−133.59	−134.93	−532.45
Total	−52.67	−92.43	−104.14	−114.30	−115.82	−479.37

Units: thousand metric tonnes CO_2_ equivalent.

## Data Availability

Not applicable.
